# Insulin-like growth factor 1 supplementation during *in vitro* maturation upregulates HRas, Cdc25, and IRS-2 expression in Kacang goat oocytes

**DOI:** 10.14202/vetworld.2026.2545-2553

**Published:** 2026-06-20

**Authors:** Widjiati Widjiati, Epy Muhammad Luqman, Eka Pramyrtha Hestianah, Viski Fitri Hendrawan, Devia Yoanita Kurniawati, Putra Aliffiansyah Farhanudin, Aulia An Nisaa Dewi, Intan Firdha Olien Noor Al Ichsan, Sirirat Rattanapuchpong

**Affiliations:** 1 Department of Veterinary Anatomy, Faculty of Veterinary Medicine, Universitas Airlangga, Surabaya, Indonesia; 2 Department of Veterinary Science, Faculty of Veterinary Medicine, Universitas Airlangga, Surabaya, Indonesia; 3 Department of Veterinary Reproduction, Faculty of Veterinary Medicine, Universitas Brawijaya, Malang, Indonesia; 4 Academic Affairs, Faculty of Veterinary Science, Chulalongkorn University, Bangkok, Thailand

**Keywords:** insulin-like growth factor 1, IRS-2, signal transduction, sustainable livestock production, zero hunger

## Abstract

**Background and Aim:**

*In vitro* maturation (IVM) is an essential assisted reproductive technology that improves oocyte competence and supports the conservation and utilization of valuable livestock genetic resources. The Kacang goat is an indigenous Indonesian breed with important economic and genetic significance. Insulin-like growth factor 1 (IGF-1) has been reported to regulate oocyte maturation through multiple intracellular signaling pathways; however, its molecular effects on maturation-associated genes in Kacang goat oocytes remain poorly characterized. This study evaluated the influence of IGF-1 supplementation during IVM on the expression of Harvey rat sarcoma viral oncogene homolog (*HRas)*, Cell division cycle 25 phosphatase (*Cdc25)*, and insulin receptor substrate-2 (IRS-2), which are associated with meiotic progression and metabolic signaling.

**Materials and Methods:**

Cumulus–oocyte complexes were collected from Kacang goat ovaries and cultured under IVM conditions either without supplementation (control) or with 100 ng/mL IGF-1. Relative expression of *HRas* and *Cdc25* was quantified using quantitative polymerase chain reaction, while IRS-2 protein expression was assessed by immunocytochemistry. Statistical comparisons between groups were performed using appropriate parametric or non-parametric tests, and correlations among molecular markers were evaluated using Spearman’s rank correlation analysis.

**Results:**

IGF-1 supplementation significantly increased the expression of both *HRas* and *Cdc25* compared with the control group (p < 0.05). The relative expression of *HRas* increased approximately 2.03-fold, whereas *Cdc25* expression increased 1.62-fold following IGF-1 treatment. Immunocytochemical analysis demonstrated significantly greater IRS-2 protein expression in IGF-1-treated oocytes than in controls (p < 0.05). Furthermore, strong positive correlations were observed among *HRas*, *Cdc25*, and IRS-2 expression levels, with the strongest association detected between *HRas* and *Cdc25* (r = 0.85). These findings indicate coordinated activation of signaling pathways involved in meiotic regulation and cellular metabolism during oocyte maturation.

**Conclusion:**

Supplementation of IVM medium with 100 ng/mL IGF-1 enhanced the expression of HRas, Cdc25, and IRS-2 in Kacang goat oocytes, suggesting activation of molecular pathways associated with meiotic resumption and metabolic regulation. These findings provide mechanistic insights into IGF-1-mediated signaling during oocyte maturation and may help optimize IVM systems for indigenous goat breeds. This improvement in reproductive biotechnology is also relevant to Sustainable Development Goal 2 (Zero Hunger), particularly in supporting sustainable livestock production, conservation of indigenous goat genetic resources, and food security. Nevertheless, further studies evaluating nuclear maturation, fertilization, and embryo developmental competence are required to confirm the functional significance of these molecular responses.

## INTRODUCTION

*In vitro* maturation (IVM) of oocytes is an important component of assisted reproductive technologies and has been widely applied to improve reproductive efficiency, support germplasm conservation, and expand the use of female gametes in livestock species. Among Indonesia’s indigenous breeds, the Kacang goat represents a valuable genetic resource with important economic and sociocultural roles. However, its long-term utilization is challenged by uncontrolled crossbreeding, suboptimal reproductive management, and the gradual erosion of valuable genetic traits. Consequently, the development of efficient reproductive biotechnologies, including optimized IVM systems, is considered essential for supporting the sustainable utilization, genetic improvement, and conservation of this indigenous goat breed [1–3].

The success of IVM depends on both intrinsic oocyte quality and the composition of the maturation medium. Numerous growth factors have been investigated to enhance oocyte developmental competence by regulating cellular metabolism, meiotic progression, and survival pathways during maturation. Among these factors, insulin-like growth factor 1 (IGF-1) has received considerable attention because of its ability to activate intracellular signaling cascades, particularly the mitogen-activated protein kinase (MAPK) and phosphoinositide 3-kinase/protein kinase B (PI3K/Protein kinase B [AKT]) pathways. These pathways regulate several key molecules involved in meiotic progression and cellular metabolism, including Harvey rat sarcoma viral oncogene homolog (*HRas)*, which participates in signal transduction associated with meiotic resumption [4, 5], cell division cycle 25 phosphatase (*Cdc25)*, which promotes G2/M transition and cell-cycle progression, and insulin receptor substrate-2 (IRS-2), which serves as a critical adaptor protein linking IGF-1 receptor activation to downstream metabolic and survival signaling pathways. Previous studies have demonstrated that IGF-1 supplementation can improve oocyte maturation and subsequent embryonic development in several mammalian species [6, 7]. Furthermore, recent findings in Kacang goat oocytes indicated that IGF-1 exerts dose-dependent biological effects during IVM, highlighting the importance of understanding the molecular mechanisms underlying its action in this species [6, 7].

Despite growing evidence supporting the beneficial role of IGF-1 during oocyte maturation, information regarding the molecular responses of Kacang goat oocytes to IGF-1 supplementation remains limited. Most previous studies have primarily focused on maturation rates, oxidative stress parameters, apoptosis-related markers, or embryonic developmental outcomes, whereas the regulation of key signaling molecules involved in meiotic control and metabolic support has received comparatively little attention. In particular, the expression patterns of *HRas* and *Cdc25*, which are associated with cell-cycle regulation and meiotic progression, together with IRS-2, a central mediator of IGF-1 signaling, have not been comprehensively investigated in Kacang goat oocytes during IVM. Consequently, the molecular pathways through which IGF-1 may influence oocyte competence in this indigenous breed remain incompletely understood. Addressing this knowledge gap is important for improving mechanistic understanding of IGF-1-mediated regulation and for developing evidence-based strategies to optimize IVM systems for Kacang goats.

Therefore, this study aimed to evaluate the effects of IGF-1 supplementation during IVM on the mRNA expression of *HRas* and *Cdc25* and the protein expression of IRS-2 in Kacang goat oocytes. By investigating these molecular markers, this study sought to elucidate the signaling mechanisms underlying IGF-1-mediated regulation during oocyte maturation and to provide molecular evidence to inform the optimization of IVM protocols for the conservation and sustainable utilization of Kacang goat genetic resources. In a broader context, optimizing IVM protocols for indigenous goat breeds also supports Sustainable Development Goal 2 (Zero Hunger) by contributing to sustainable livestock production, preservation of local genetic resources, and improvement of reproductive efficiency in small ruminants.

## MATERIALS AND METHODS

### Ethical approval

The experimental procedures and use of animal-derived biological materials in this study were reviewed and approved by the Animal Care and Use Committee, Faculty of Veterinary Medicine, Universitas Airlangga, Surabaya, Indonesia (Approval No. 1.KEH.146.10.2024). Ovarian samples were obtained exclusively from clinically healthy Kacang goats (Capra hircus) slaughtered for commercial purposes at a licensed local abattoir. No animals were purchased, housed, handled, restrained, or euthanized specifically for this research.

Ovary collection was performed immediately after slaughter by trained personnel in accordance with abattoir regulations and institutional biosafety guidelines. The collection procedure did not interfere with standard slaughterhouse operations and involved only the retrieval of reproductive tissues that would otherwise have been discarded as biological waste. Therefore, no additional stress, pain, discomfort, or invasive procedures were imposed on the animals beyond routine commercial slaughter practices.

All biological samples were transported, processed, and handled in accordance with the ethical standards established by the Faculty of Veterinary Medicine, Universitas Airlangga. Laboratory procedures involving oocyte recovery, *IVM*, molecular analysis, and immunocytochemical examination were conducted in accordance with institutional guidelines for the responsible use of animal-derived materials in scientific research. The study complied with applicable national regulations and internationally accepted principles for animal welfare, ethical research conduct, and the reduction of animal use in research.

Because the study used post-slaughter reproductive tissues collected from animals slaughtered for food production and did not involve any live-animal experimental interventions, additional animal welfare concerns were minimized while ensuring the study’s scientific objectives were achieved.

### Study period and location

The study was conducted from July to December 2025. Oocyte collection, processing, and IVM procedures were performed at the Laboratory of Veterinary Reproduction, Faculty of Veterinary Medicine, Universitas Airlangga, Surabaya, Indonesia. Gene-expression analysis by quantitative polymerase chain reaction (qPCR) was conducted at the Institute of Tropical Diseases, Universitas Airlangga, Surabaya, Indonesia. Immunocytochemical analysis was performed at the Pathology Laboratory, Faculty of Veterinary Medicine, Universitas Airlangga, Surabaya, Indonesia.

### Study design

This experimental study employed a completely randomized design consisting of two treatment groups. The control group consisted of oocytes matured in standard IVM medium without IGF-1 supplementation, whereas the treatment group consisted of oocytes matured in the same medium supplemented with 100 ng/mL IGF-1. Following maturation, *HRas* and *Cdc25* expression were evaluated at the messenger RNA (mRNA) level by qPCR, whereas IRS-2 expression was assessed at the protein level by immunocytochemistry (ICC). Correlations among *HRas*, *Cdc25*, and IRS-2 expression were subsequently analyzed.

### Collection and transportation of ovarian samples

Ovaries were collected from healthy, non-pregnant female Kacang goats aged 6–12 months at a local slaughterhouse. The ovaries were removed between 05:00 and 06:00 and immediately placed in prewarmed 0.9% saline solution containing streptomycin (Sigma-Aldrich, St. Louis, MO, USA). Samples were transported to the laboratory within 2 h using a thermally insulated container (Akebonno, Jakarta, Indonesia). Upon arrival, the ovaries were washed three times with (PBS, pH 7.4; Thermo Fisher Scientific, Waltham, MA, USA) to remove blood and tissue debris.

### Oocyte selection and IVM

Follicular contents were aspirated using a 10 mL syringe fitted with an 18-gauge needle. Only cumulus–oocyte complexes (COCs) possessing at least three compact layers of cumulus cells and homogeneous ooplasm were selected under an inverted microscope, whereas degenerated or atretic oocytes were excluded.

Selected COCs were randomly allocated into two experimental groups. The control group was cultured in standard maturation medium, whereas the treatment group was cultured in the same medium supplemented with 100 ng/mL IGF-1. The maturation medium consisted of TCM-199 (Thermo Fisher Scientific) supplemented with 10% fetal bovine serum (Sigma-Aldrich, St. Louis, MO, USA), 10 μg/mL follicle-stimulating hormone (FSH; Sigma-Aldrich, St. Louis, MO, USA), 10 μg/mL luteinizing hormone (LH; Sigma-Aldrich), and 1% penicillin-streptomycin (Thermo Fisher Scientific). COCs were cultured in groups of 10 oocytes per 30 μL droplet under mineral oil (Sigma-Aldrich) for 22 h at 38.5°C in a humidified atmosphere containing 5% CO_2_.

### RNA extraction and qPCR analysis

Total RNA was extracted from pooled matured oocytes using the PureLink RNA Mini Kit (Invitrogen, Carlsbad, CA, USA), followed by purification using the RNeasy Micro Kit (Qiagen, Hilden, Germany). RNA concentration was measured using a Qubit Fluorometer (Thermo Fisher Scientific), and complementary DNA (cDNA) was synthesized using the iScript cDNA Synthesis Kit (Bio-Rad Laboratories, Hercules, CA, USA). The relative expression of *HRas* and *Cdc25* was determined using qPCR, with Beta-actin (*ACTB)* used as the reference gene (Table 1). Amplification was performed using SYBR Green chemistry in a LightCycler 480 II Real-Time PCR System (Roche Diagnostics, Mannheim, Germany). Relative expression levels were calculated using the 2^−ΔΔCt method. IRS-2 was not analyzed at the mRNA level in this study and was assessed only at the protein level by ICC.

**Table 1:** Primers used for quantitative polymerase chain reaction analysis of *HRas*, *Cdc25c*, and *ACTB.*

Target gene	Primer sequence (5′→3′)	Melting temperature	Length	Annealing temperature
*HRas*	F: 5′-GCCATCAACCACACCAAGTCC-3′	62.0°C	20 bp	58°C
	R: 5′-GGAGCTGCAGCCAGAGCCAGA-3′	65.1°C	22 bp	
*Cdc25c*	F: 5′-TGGAGTCTACAGGACCTGAGCAA-3′	63.8°C	23 bp	59.3°C
	R: 5′-TGGGACTGCCCAGATGTTTCA-3′	61.3°C	22 bp	
*ACTB*	F: 5′-GCAAGGACCTTTACGCCAAC-3′	59.8°C	20 bp	57.4°C
	R: 5′-CTTGATCTTCATCGTGCTGGG-3′	61.4°C	21 bp	

### ICC

IRS-2 expression was evaluated at the protein level by ICC. Matured oocytes were fixed in methanol:acetic acid (3:1), permeabilized with 0.025% trypsin, and blocked using Ultra V Block (Thermo Fisher Scientific). Samples were incubated with anti-IRS-2 primary antibody (Solarbio, Beijing, China; Cat. No. K005574P), followed by a biotinylated secondary antibody and streptavidin-horseradish peroxidase. Immunoreactivity was visualized using 3,3′-diaminobenzidine as the chromogen and methylene green as the counterstain.

Oocytes were examined under a light microscope at 400× magnification (Olympus, Japan). The immunoreactive score was determined from the product of staining intensity and the proportion of positive staining. A total of six slides per group were evaluated, with five oocytes examined per slide, for a total of 30 oocytes analyzed per group.

### Statistical analysis

Data for *HRas* and *Cdc25* expression are presented as mean ± standard error of the mean. Data normality and homogeneity were assessed using the Shapiro–Wilk and Levene tests, respectively. Comparisons between groups were performed using the independent-samples t-test for normally distributed data and the Mann–Whitney U test for nonparametric data. Because immunoreactive score data were analyzed nonparametrically, IRS-2 protein expression results are presented as mean rank values. Correlations among *HRas, Cdc25*, and *IRS-2* expression were evaluated using Spearman’s rank correlation analysis. Differences were considered statistically significant at p < 0.05.

## RESULTS

### Relative expression of *HRas* and *Cdc25*

Quantitative polymerase chain reaction analysis demonstrated that IGF-1 supplementation significantly increased the relative expression of *HRas* and *Cdc25* compared with the control group (p < 0.05) (Table 2). The mean relative expression of *HRas* increased from 4.91 ± 0.69 in the control group to 9.97 ± 0.48 in the IGF-1-treated group, whereas *Cdc25* expression increased from 0.73 ± 0.04 to 1.18 ± 0.08. These findings corresponded to 2.03-fold and 1.62-fold increases in the expression of *HRas* and *Cdc25*, respectively, following IGF-1 supplementation.

**Table 2:** Relative expression of *HRas* and *Cdc25* in Kacang goat oocytes cultured with or without IGF-1.

Group	n	*HRas* (Mean ± SEM)	*Cdc25* (Mean ± SEM)
Control	6	4.91 ± 0.69ᵃ	0.73 ± 0.04ᵃ
IGF-1 (100 ng/mL)	6	9.97 ± 0.48ᵇ	1.18 ± 0.08ᵇ

Different superscripts within a column indicate significant differences (p < 0.05).

**Figure 1:**
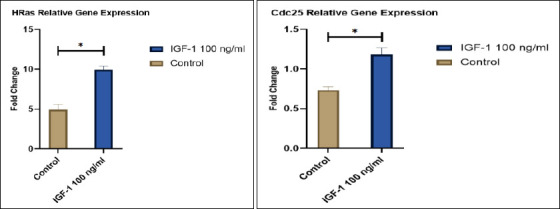
Fold-change in the relative expression of *HRas* and *Cdc25* in IGF-1-treated oocytes relative to the control group.

As illustrated in Figure 1, IGF-1 supplementation increased the expression of both *HRas* and *Cdc25*. The increase in *HRas* expression was more pronounced than that observed for *Cdc25*, suggesting a stronger transcriptional response of *HRas* to IGF-1 supplementation during IVM.

### IRS-2 protein expression

Immunocytochemical analysis revealed stronger IRS-2 immunoreactivity in the IGF-1-treated group than in the control group (Table 3). Because immunoreactive score data were analyzed using nonparametric methods, the results are presented as mean rank values. The IGF-1-treated group exhibited a significantly higher mean rank than the control group (p < 0.05), indicating enhanced IRS-2 protein expression following IGF-1 supplementation.

**Table 3:** IRS-2 protein expression in Kacang goat oocytes evaluated by immunocytochemistry.

Group	n	IRS-2 expression (Mean rank)
Control	6	6.63ᵃ
IGF-1 (100 ng/mL)	6	10.60ᵇ

Different superscripts indicate significant differences (p < 0.05). Six slides were evaluated per group, with five oocytes examined per slide.

**Figure 2:**
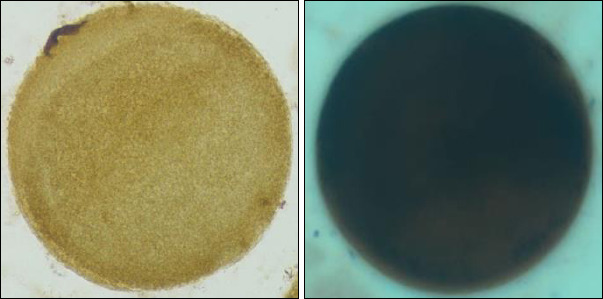
IRS-2 protein expression in Kacang goat oocytes evaluated by immunocytochemistry (400× magnification). The control group exhibited light brown cytoplasmic staining, whereas the IGF-1-treated group showed intense brown cytoplasmic staining, indicating increased IRS-2 protein expression.

Figure 2 illustrates the immunocytochemical localization of IRS-2 in Kacang goat oocytes. Oocytes from the control group exhibited light brown cytoplasmic staining, whereas those from the IGF-1-treated group showed intense brown cytoplasmic staining, indicating greater IRS-2 protein expression.

### Correlation among *HRas, Cdc25*, and IRS-2

**Figure 3:**
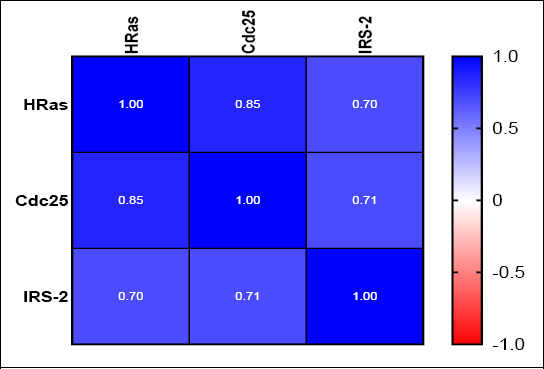
Heatmap showing correlation coefficients among *HRas*, *Cdc25*, and IRS-2 expression levels. Positive correlations are represented by a gradient blue scale, indicating coordinated gene and protein expression following IGF-1 supplemen-tation.

Spearman’s rank correlation analysis revealed strong and statistically significant positive correlations among the expression levels of *HRas*, *Cdc25*, and IRS-2 (Figure 3). The strongest correlation was observed between *HRas* and *Cdc25* (r = 0.85), followed by *Cdc25* and IRS-2 (r = 0.71), and *HRas* and IRS-2 (r = 0.70). These findings suggest coordinated regulation of molecular pathways associated with meiotic progression and metabolic signaling in response to IGF-1 supplementation.

## DISCUSSION

### IGF-1 regulates molecular pathways associated with oocyte maturation

IGF-1 is a well-recognized growth factor involved in folliculogenesis, granulosa cell function, and oocyte maturation. Through activation of intracellular signaling pathways, particularly MAPK and PI3K/AKT, IGF-1 contributes to meiotic regulation, metabolic support, and cellular survival during oocyte maturation [5–11]. In the present study, IGF-1 supplementation during IVM significantly increased the expression of *HRas*, *Cdc25*, and IRS-2, indicating activation of molecular pathways associated with cell-cycle progression and intracellular signaling in Kacang goat oocytes.

### Increased *HRas* and *Cdc25* expression following IGF-1 supplementation

The increased expression of *HRas* observed in this study is consistent with activation of MAPK-related signaling during oocyte maturation. As a member of the RAS family, *HRas* functions upstream of the RAF-MEK-ERK signaling cascade and participates in signal transduction processes associated with cell proliferation, differentiation, and cell-cycle regulation [12]. In oocytes, MAPK signaling contributes to meiotic progression and the regulation of maturation-associated transcripts [13]. Therefore, the increased expression of *HRas* following IGF-1 supplementation may indicate enhanced activation of signaling pathways associated with meiotic resumption and oocyte competence.

Similarly, the increase in *Cdc25* expression suggests enhanced regulation of the G2/M transition. Cdc25 phosphatases activate cyclin-dependent kinase complexes that are required for M-phase entry and cell-cycle progression [14, 15]. Consequently, the concurrent upregulation of *HRas* and *Cdc25* in the IGF-1-treated group suggests that IGF-1 may promote molecular events associated with meiotic progression during IVM.

However, direct maturation endpoints such as germinal vesicle breakdown, metaphase II attainment, polar body extrusion, and cumulus expansion were not evaluated in the present study. Therefore, the observed increases in *HRas* and *Cdc25* expression should be interpreted as molecular evidence associated with maturation-related signaling rather than direct evidence of improved functional oocyte maturation.

### Enhanced IRS-2 expression indicates activation of IGF-1 signaling

The elevated IRS-2 protein expression further supports the gene-expression findings. IRS-2 is a major adaptor molecule in insulin and IGF-1 receptor signaling and serves as an important intermediary linking receptor activation to the PI3K/AKT pathway [1, 2, 16]. This signaling axis has been implicated in the regulation of cellular metabolism, survival, and responsiveness in reproductive cells, including oocytes and granulosa cells [2, 10, 11]. Accordingly, the increased IRS-2 immunoreactivity observed in the IGF-1-treated group may reflect enhanced responsiveness of Kacang goat oocytes to IGF-1-mediated signaling.

The positive correlations observed among *HRas*, *Cdc25*, and IRS-2 further suggest coordinated regulation of pathways associated with meiotic control and metabolic support. Nevertheless, these correlations should not be interpreted as evidence of direct causal relationships. Additional mechanistic studies involving pathway inhibition, gene knockdown approaches, or phosphorylation-based analyses are necessary to verify the functional interactions among these signaling components.

### Comparison with previous studies

The present findings are consistent with previous reports investigating the effects of IGF-1 during oocyte maturation. A related study by *Widjiati et al*. [17] demonstrated that IGF-1 supplementation exerted dose-dependent effects on oocyte quality during IVM, with different concentrations producing distinct effects on oxidative stress and apoptotic responses. These observations indicate that the biological effects of IGF-1 are not necessarily linear. Consequently, the concentration of 100 ng/mL used in the present study should be regarded as a defined experimental concentration for evaluating molecular responses rather than a definitively optimized concentration for improving overall oocyte competence.

Previous studies in goats and other mammalian species have also reported that IGF-1 supplementation may improve maturation-related responses and early embryonic development, although the effective concentration and biological outcomes vary according to species, culture systems, and experimental conditions [6, 7, 10, 18]. Therefore, the present study provides valuable molecular information on the response of Kacang goat oocytes to IGF-1 and highlights the need for additional dose-response investigations.

### Implications for reproductive biotechnology in Kacang goats

The upregulation of *HRas*, *Cdc25*, and IRS-2 suggests that IGF-1 may contribute to the establishment of a molecular environment favorable for meiotic and metabolic regulation during IVM. These findings may be relevant to the refinement of reproductive biotechnologies for indigenous breeds such as the Kacang goat, which represents an important local genetic resource [17, 19].

Nevertheless, the present data do not directly demonstrate improvements in cleavage rate, embryo yield, blastocyst formation, fertilization success, or overall developmental competence. Accordingly, the implications of this study should be limited to molecular signaling events associated with IVM, rather than to broader reproductive outcomes.

Optimizing IVM systems in indigenous livestock breeds may also indirectly contribute to sustainable livestock production and related initiatives associated with Sustainable Development Goal 2 (Zero Hunger). However, this potential relevance should be interpreted cautiously because the present study did not evaluate field-level productivity, embryo production efficiency, or food-system outcomes.

### Limitations of the study and future research directions

Several limitations should be acknowledged when interpreting the present findings. First, direct maturation outcomes, including metaphase II rate, polar body extrusion, cumulus expansion, and degeneration rate after 22 h of IVM, were not assessed. Second, embryo developmental outcomes, including cleavage rate and blastocyst formation, were not evaluated; therefore, developmental competence could not be directly confirmed [20]. Third, only a single IGF-1 concentration was examined, limiting the interpretation of dose-dependent biological responses [17]. Finally, the analysis was restricted to selected gene and protein markers and did not include post-translational validation or functional interrogation of the signaling pathways involved.

Future studies should integrate molecular analyses with direct assessments of oocyte maturation, fertilization success, embryo development, and dose-response evaluation. Investigations involving pathway-specific inhibitors, phosphorylation assays, and functional validation approaches would provide a more comprehensive understanding of the mechanisms through which IGF-1 regulates oocyte maturation and developmental competence in Kacang goats.

## CONCLUSION

Supplementation with IGF-1 during IVM increased *HRas* and *Cdc25* mRNA expression and increased IRS-2 protein expression in Kacang goat oocytes. The observed upregulation of these markers, together with the significant positive correlations among *HRas*, *Cdc25*, and IRS-2, suggests activation of molecular pathways associated with meiotic regulation, intracellular signaling, and metabolic support during oocyte maturation. Among the evaluated markers, *HRas* exhibited the greatest response to IGF-1 supplementation, indicating a potentially important role of MAPK-related signaling in the molecular regulation of Kacang goat oocyte maturation.

These findings provide novel molecular evidence on the response of Kacang goat oocytes to IGF-1 supplementation and contribute to a better understanding of the signaling events that occur during IVM in this indigenous breed. From a practical perspective, the results may support future efforts to refine IVM systems and reproductive biotechnologies aimed at the conservation, genetic improvement, and sustainable utilization of Kacang goat genetic resources.

A major strength of this study is the combined evaluation of gene and protein markers associated with IGF-1 signaling, allowing a more comprehensive assessment of molecular responses during IVM. However, several limitations should be considered. The study did not evaluate direct maturation outcomes such as metaphase II attainment, polar body extrusion, or cumulus expansion. In addition, embryo developmental competence, including cleavage and blastocyst formation, was not assessed. The investigation was limited to a single IGF-1 concentration and a selected set of molecular markers without functional pathway validation.

Future studies should integrate molecular analyses with assessments of nuclear and cytoplasmic maturation, fertilization success, embryo development, and dose-response evaluation of IGF-1 supplementation. Functional studies involving pathway inhibition, phosphorylation analysis, and additional signaling markers would further clarify the mechanisms through which IGF-1 regulates oocyte maturation. Overall, IGF-1 supplementation at 100 ng/mL promoted molecular changes associated with oocyte maturation in Kacang goat oocytes and represents a promising approach for further optimization of IVM systems in this important indigenous goat breed. In a broader context, optimization of IVM systems for Kacang goat oocytes is relevant to Sustainable Development Goal 2 (Zero Hunger), as it may support sustainable livestock production, preservation of indigenous goat genetic resources, and improvement of reproductive efficiency in small ruminants.

## DATA AVAILABILITY

The data generated during the study are included in the manuscript.

## AUTHORS’ CONTRIBUTIONS

WW and EML: Conceptualization, methodology, supervision, validation, manuscript review and editing, and final approval of the manuscript. EPH: Methodology, supervision, validation, manuscript review and editing, and final approval of the manuscript. VFH: Methodology, data analysis, interpretation of results, manuscript drafting, manuscript review and editing, and final approval of the manuscript. DYK: Investigation, data collection, laboratory work, data analysis, manuscript drafting, and final approval of the manuscript. PAF: Investigation, oocyte collection and *IVM* procedures, sample preparation, manuscript drafting, and final approval of the manuscript. AAND and IFONAI: Investigation, laboratory assistance, data collection, sample preparation, manuscript drafting, and final approval of the manuscript. SR: Methodology support, scientific review, manuscript review and editing, and final approval of the manuscript. All authors have read and approved the final manuscript.
